# Fatal Cases of Influenza A(H3N2) in Children: Insights from Whole Genome Sequence Analysis

**DOI:** 10.1371/journal.pone.0033166

**Published:** 2012-03-06

**Authors:** Monica Galiano, Benjamin F. Johnson, Richard Myers, Joanna Ellis, Rod Daniels, Maria Zambon

**Affiliations:** 1 Microbiology Services, Health Protection Agency, London, United Kingdom; 2 Section of Virology, Faculty of Medicine, Imperial College London, St. Mary's Campus, London, United Kingdom; 3 Division of Virology, MRC National Institute for Medical Research, London, United Kingdom; University of Hong Kong, Hong Kong

## Abstract

During the Northern Hemisphere winter of 2003–2004 the emergence of a novel influenza antigenic variant, A/Fujian/411/2002-like(H3N2), was associated with an unusually high number of fatalities in children. Seventeen fatal cases in the UK were laboratory confirmed for Fujian/411-like viruses. To look for phylogenetic patterns and genetic markers that might be associated with increased virulence, sequencing and phylogenetic analysis of the whole genomes of 63 viruses isolated from fatal cases and non fatal “control” cases was undertaken. The analysis revealed the circulation of two main genetic groups, I and II, both of which contained viruses from fatal cases. No associated amino acid substitutions could be linked with an exclusive or higher occurrence in fatal cases. The Fujian/411-like viruses in genetic groups I and II completely displaced other A(H3N2) viruses, but they disappeared after 2004. This study shows that two A(H3N2) virus genotypes circulated exclusively during the winter of 2003–2004 in the UK and caused an unusually high number of deaths in children. Host factors related to immune state and differences in genetic background between patients may also play important roles in determining the outcome of an influenza infection.

## Introduction

Influenza viruses are a common cause of human respiratory infections [1]. Epidemics occur every year during the winter seasons in the Northern and Southern Hemispheres and result in considerable morbidity and mortality. Disease severity is greatest in the elderly, in infants and in people with certain chronic diseases. An average of 12,554 deaths occurred in England and Wales during annual influenza epidemics between 1990–2000 [2].

Acquisition of point mutations in the haemagglutinin glycoprotein of influenza A virus leads to continuous antigenic change, a process called ‘antigenic drift’. This results in continuous replacement of circulating viruses with new variants which are able to re-infect hosts despite their immunity to antigenic variants that circulated previously. In humans, A(H3N2) viruses are considered to evolve faster than the A(H1N1) subtype [3], [4]. Every three to eight years, predominant A(H3N2) viruses are replaced by a novel antigenic variant, prompting an update of the recommended influenza vaccine strain [5]. During the 2002–2007 period, the A(H3N2) component of the vaccine was updated four times [6].

A(H3N2) viruses are associated with increased morbidity and mortality [7]. The Northern Hemisphere season of 2003–2004 was characterised by the emergence of an antigenic drift variant, A/Fujian/411/2002, which completely displaced the previously circulating variant, A/Panama/2007/99. Initial circulation of the Fujian/411-like variants in the UK and the US was accompanied by an unusually high number of influenza-associated fatalities in children [8], [9]. Seventeen such laboratory-confirmed influenza cases were reported in the UK during 2003–2004. Clinical and pathological findings identified no recognised pre-existing risk factors for severe influenza illness in 88% of the fatal cases and only 18% presented secondary bacterial infections. Serological and community morbidity studies showed increased susceptibility in the youngest age groups [8]. This posed the question of whether intrinsic virus virulence or underlying host susceptibility was more important in determining a fatal outcome. The aim of this study was to identify any genetic markers of virulence in Fujian/411-like influenza A viruses from 2003–2004 by sequencing whole genomes of viruses isolated from fatal and non-fatal paediatric infections in the UK. Genetic information was used to determine if virus mutations were associated with fatal outcome by comparison with genetic features of viruses from previous and subsequent influenza seasons and viruses from the same season elsewhere.

## Results

### Whole-genome analysis of influenza sequences from fatal and non-fatal cases

Sequences of the complete coding regions of influenza whole genomes were obtained from original respiratory material and/or viruses isolated from 12/17 fatal cases (Table S2). The remaining 5 fatal cases had HA1 fragment sequences available from original material only. Three viruses from non-fatal, adult contacts of fatal case #6 (A/England/740/2003) were also isolated and genome-sequenced. Further genomes were sequenced from 51 viruses obtained from non-fatal control cases. Where samples were available (14/63), sequencing was performed both from viral isolate and original material.

Given that the focus of our study was on detecting mutations that could be associated with more severe infections, we first sought to establish whether genetic changes could have been induced during the adaptation to cell culture isolation. Comparison of sequences showed no genetic changes generated through virus culture for any of the 14 original material/isolate pairs, except for virus A/England/484/03 (control case) which showed a mutation G720A in the haemagglutinin (HA) gene sequence from the viral isolate (corresponding to V242I in the deduced HA protein). Overlapping peaks were observed at that position in the HA of the isolate sequence (data not shown), suggesting the presence of a mixture of variants or quasispecies which may have been differentially selected through culture.

**Figure 1:**
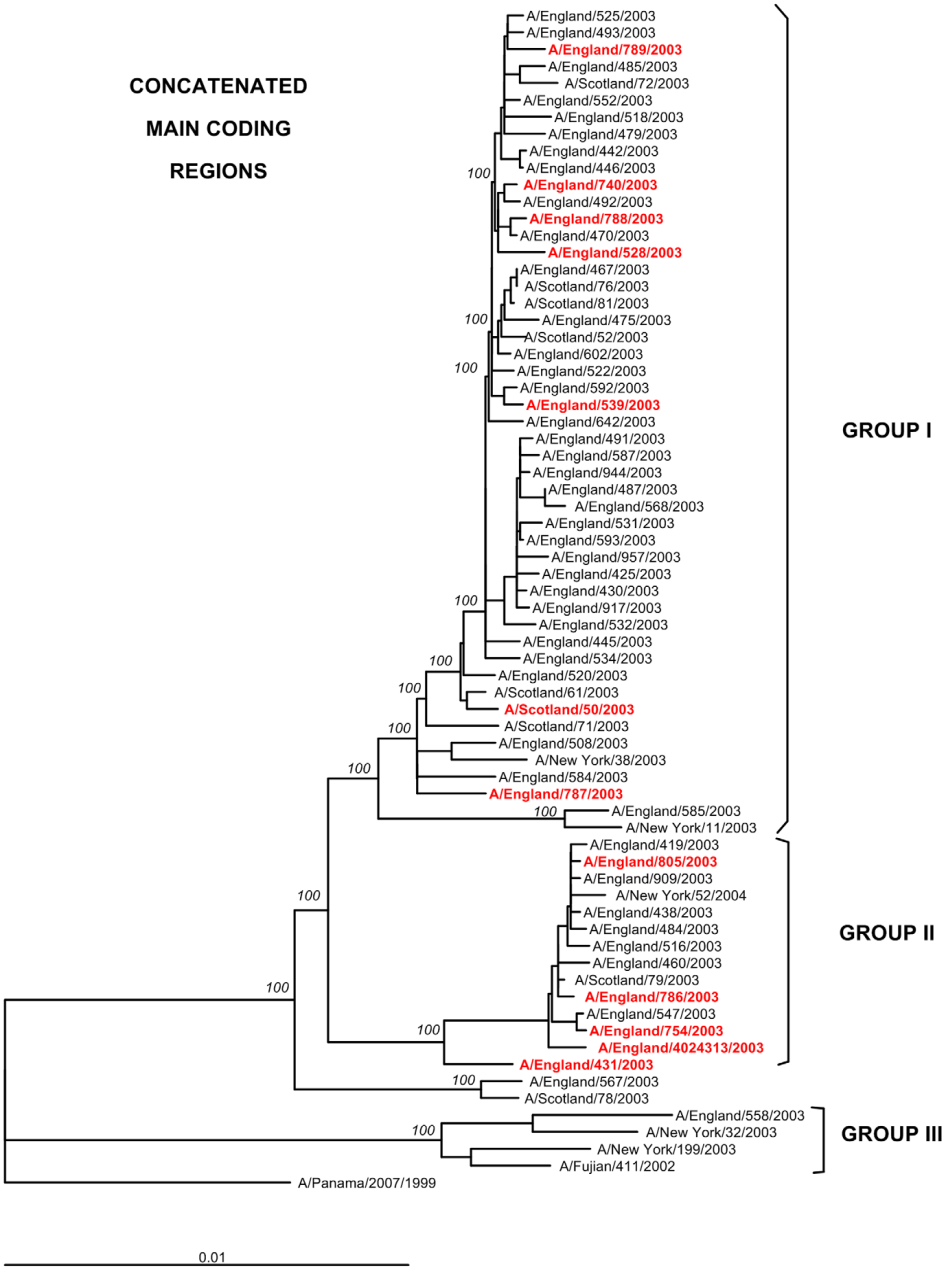
Phylogenetic relationships of the concatenated main coding regions of influenza A(H3N2) viruses isolated in the UK during 2003–2004. Sequences from reference viruses A/Panama/2007/99 and A/Fujian/411/2002 were included in the dendrograms. For the purposes of comparison, the dendrograms also included sequences from A/New York/38/2003, A/New York/11/2003, A/New York/32/2003, A/New York/199/2003 and A/New York/52/2004. All phylogenies were rooted using A/Panama/2007/99 as an outgroup. Branch lengths are drawn to scale. Viruses isolated from fatal cases are displayed in red type. Bootstrap values (>70%) are displayed on the nodes. Genetic groups I and II are indicated with brackets. Amino acid substitutions characterising these groups are annotated on the nodes.

A phylogenetic tree was inferred using the concatenated sequences of the main coding regions of 70 viruses (Figure 1 new). For the purpose of comparison, we also included genome sequences representing the main clusters seen in A(H3N2) viruses from New York during the season of 2003–2004 [10]. Virus sequences from the adult contacts of fatal case #6 were not included in the trees to avoid statistical bias. The analysis revealed that UK A(H3N2) viruses from 2003–2004 clustered within two genetic groups, named I and II. Two UK viruses, A/England/567/2003 and A/Scotland/78/2003 grouped in a single branch, external to both genetic groups I and II. A third minor genetic group (III) outgrouping all the others included a single UK strain, A/England/558/2003 (control case), together with A/Fujian/411/2002 and two viruses from New York, All viruses isolated from fatal cases were interspersed with control viruses within genetic groups I and II, with no evidence for clustering that could suggest genetic differences specific to the fatal cases viruses.

**Figure 2 pone-0033166-g002:**
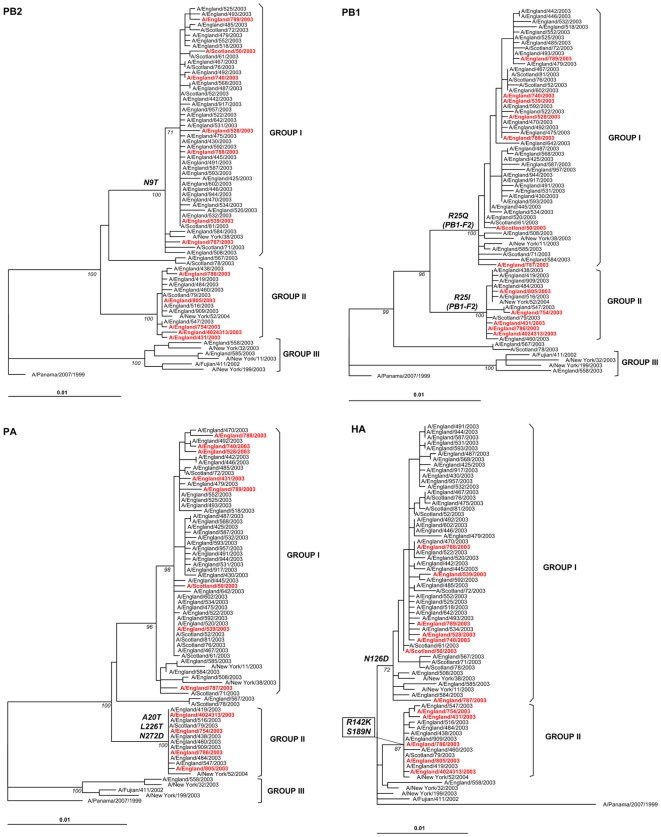
Phylogenetic relationships of the main coding regions of PB2, PB1, PA & HA of influenza A(H3N2) viruses isolated in the UK during 2003–2004. Details are similar to those described in Figure 1 footnote.

**Figure 3:**
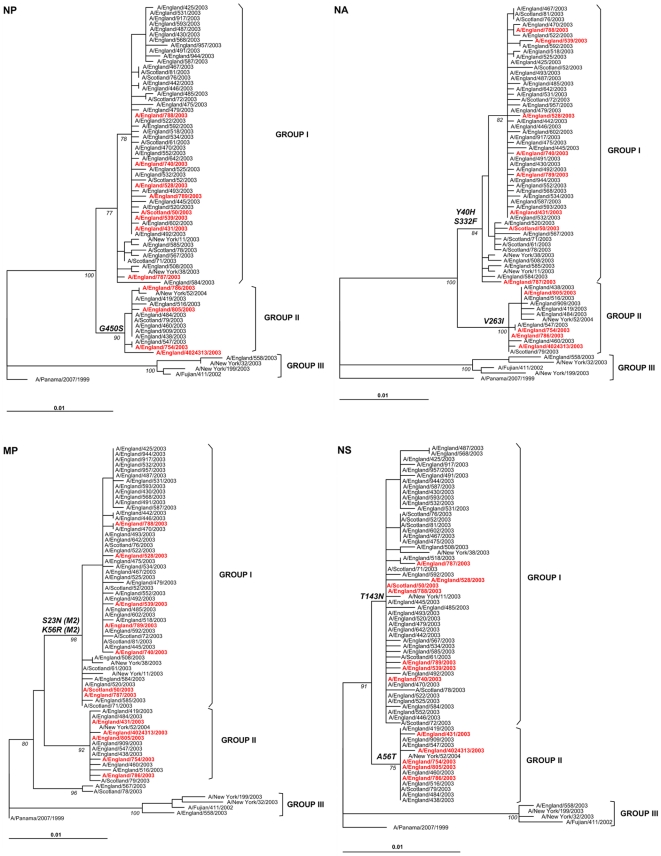
Phylogenetic relationships of the main coding regions of NP, NA, M & NS of influenza A(H3N2) viruses isolated in the UK during 2003–2004. Details are similar to those described in Figure 1 footnote.

Next, we constructed phylogenetic trees of the main coding regions for each gene Figures 2 & 3). The topology of the individual gene trees was very similar to the concatenated tree, with distinctive separation of viruses into three genetic groups, except in the HA phylogeny where the branch corresponding to genetic group III was not evident.

### Evidence for gene reassortment

In general, there was a clear co-segregation of genes from each virus, as shown by similar phylogenetic topologies, with some exceptions: a) genes NA, NP and PA from fatal case A/England/431/2003 (fatal case #4) clustered within genetic group I, whereas the rest of its genes fell within genetic group II; b) genes from control case A/England/585/2003 fell into genetic group I, except for PB2 which fell within genetic group III; c) while most genes of A/England/558/2003 fell into genetic group III, the HA fell in a branch whose closest relationship was with genetic group II; d) genes NP, NA, NS and HA from control viruses A/England/567/2003 and A/Scotland/78/2003 clearly clustered within genetic group I, while PB2 and PA fell in a separate distinct branch that clustered with genetic group I (albeit with <70% bootstrap values) - sequences from their PB1 and M genes grouped in a single branch, external to both genetic groups I and II. These phylogenetic incongruencies suggest the occurrence of reassortment events for all these viruses. In addition, all the full genome concatenated sequences were tested for the presence of mosaicism using bootscanning and informative sites analysis in order to formally identify recombination breakpoints (Figure S1 & Text S1). The analysis confirmed the presence of reassortment (recombination in a concatenated genome) and the breakpoints detected were consistent with the genes involved in the reassortment for each in the above mentioned viruses.

### Amino acid substitutions associated with a fatal outcome

Given the presence of these incongruencies in the phylogenetic topologies, the number of viruses clustering into each genetic group varied slightly for each dendrogram. Genetic group II contained 4–5 out of 12 fatal cases (33–42%), depending on the gene, but only 8 out of 51 non-fatal cases (16%). The difference between these percentages was not significant (Fisher's exact test values ranging from p = 0.107 to p = 0.234, depending on the gene segment).

We next looked at the inferred protein sequences (including PB1-F2, NS2 & M2) from the gene alignments to determine if specific amino acid substitutions could be consistently associated with a fatal outcome. None of the amino acid polymorphisms present within the whole genome was uniquely or consistently present in viruses from the fatal cases. Observed signature substitutions that differentiated genetic groups I and II, as annotated in Figures 2 & 3, were present in all viruses in these clusters irrespective of the clinical outcome.

An epidemiological cluster was defined for fatal case #6, A/England/740/2003: A/England/741/2003 was recovered from a family contact (sibling of unknown age), while A/England/752/2003 and A/England/753/2003 were isolated from adult healthcare workers who had been in contact with the index fatal case. The contact cases developed influenza-like illness with no severe complications and fully recovered. Comparison of the coding regions of the viruses recovered from the A/England/740/2003 case contacts showed that nucleotide coding sequences were identical for all genes despite the differences in disease severity observed.

### Glycosylation of haemagglutinin

It has been postulated that, for A(H3N2) viruses, an increase in the number of potential sites of N-linked glycosylation in the HA protein resulted in decreased virulence [11]. Ten potential N-glycosylation sites were identified along the full length HA of both control and fatal case viruses, at the following residues: 8, 22, 63, 126, 133, 144, 165, 246, 285 (all in HA1) and 483 (in HA2). The potential glycosylation sites were conserved among the viruses with the exception of position 126, which was absent in UK viruses belonging to genetic group I. These findings were related to phylogenetic clustering and do not associate with a fatal outcome.

### Worldwide prevalence of viruses belonging to UK genetic groups I and II

We next attempted to assess the frequency with which viruses isolated worldwide during 2003–2004 clustered with UK viruses belonging to genetic groups I and II. However, when trying to do so by using whole genome sequences available in GenBank, we found 413 genomes from A(H3N2) influenza viruses from 2003–2004, strongly biased towards viruses recovered in the US, Australia and New Zealand. We therefore performed this ‘prevalence’ phylogenetic analysis with a larger, worldwide-representative dataset of 1,893 HA1 sequences (positions 346 to 866 of the HA ORF, encompassing the main antigenic sites of the HA1 glycoprotein) including the UK viruses and all A(H3N2) viruses isolated in 2003–2004 as available in GenBank [12]. This allowed us to include those obtained from original material for the five fatal cases for which full genome sequences were not available.

**Figure 4:**
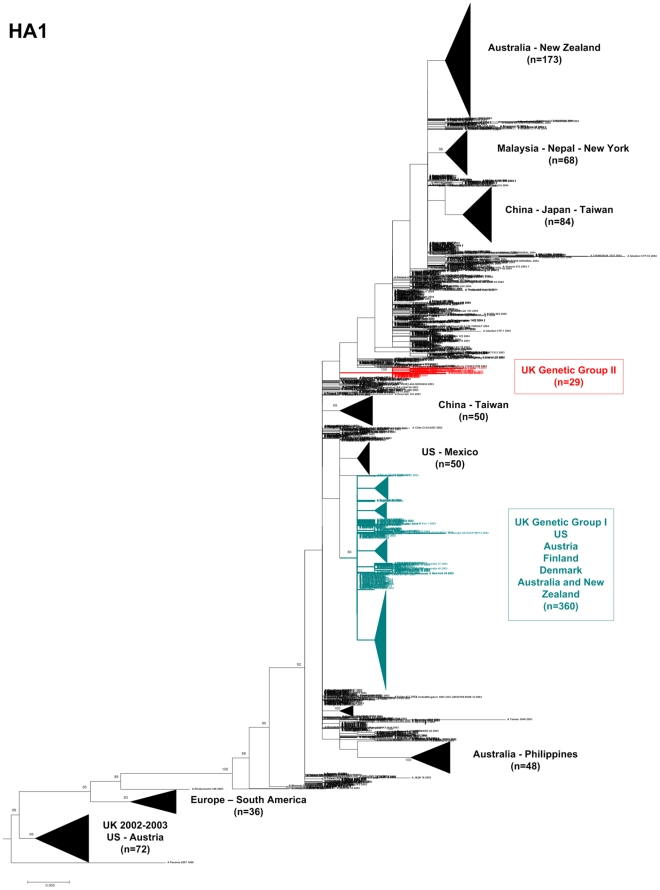
Phylogenetic relationships of partial sequences (nucleotides 346 to 866) of the HA1 coding fragment for A(H3N2) viruses isolated in 2003–2004 in the UK and worldwide. Genetic groups where viruses from the UK clustered are highlighted in green (I) and red (II). Some branches were condensed for clarity purposes; adjacent legends indicate the country/region where the majority of viruses within a cluster were isolated and the number of viruses included. These clusters also included sporadic viruses from other countries which were not detailed. Sequences from reference viruses A/Panama/2007/99 and A/Fujian/411/2002 were included in the phylogeny. The ML phylogeny was rooted using A/Panama/2007/99 as an outgroup. Branch lengths are drawn to scale. Bootstrap values (>70%) are displayed on the nodes.

The phylogenetic relationships in this dendrogram showed that there was clear evidence of geographical clustering, where most clusters showed predominance of viruses from the same region or neighbouring countries (Figure 4). However, in each cluster there were also sporadic viruses from other regions (not detailed in the figure). Genetic group I UK viruses formed part of a large cluster (highlighted in green) with a robust bootstrap value (80%) comprising 360 viruses from the rest of Europe (Austria, Finland and Denmark), North America (US) and Oceania (Australia and New Zealand). There were at least four subclusters within this group (condensed for clarity): i) US+UK viruses; ii) UK+Finland+Denmark; iii) Austria; iv) Australia+New Zealand. Genetic group I UK viruses were also represented in single branches within this large cluster.

In contrast, genetic group II UK viruses clustered in a small branch of 29 viruses out of 1,893 with a 100% bootstrap (highlighted in red). While 15 of these were UK viruses (including 6 from fatal cases) the remaining 14 were from the US (n = 5) and some European countries (France (2), Romania (1), Italy (1), Sweden (1), Denmark (2) and Norway (2)). The prevalence of genetic group II viruses worldwide, based on the total number of HA1 sequences from 2003–2004 available in GenBank, was only 1.5%, compared to a higher prevalence of 22% (n = 15/68) in the UK.

### Evolution of A(H3N2) influenza viruses after 2003–2004 in the UK

To determine the prevalence and/or survival of genetic groups I and II in subsequent influenza seasons, we analysed our initial set of viruses in a larger dataset of UK sequences encompassing influenza seasons from 2002–2003 to 2007–2008, using the same HA1 fragment selected to construct the phylogeny presented in Figure 4, again including the sequences from the five ‘HA1 only’ fatal cases.

**Figure 5:**
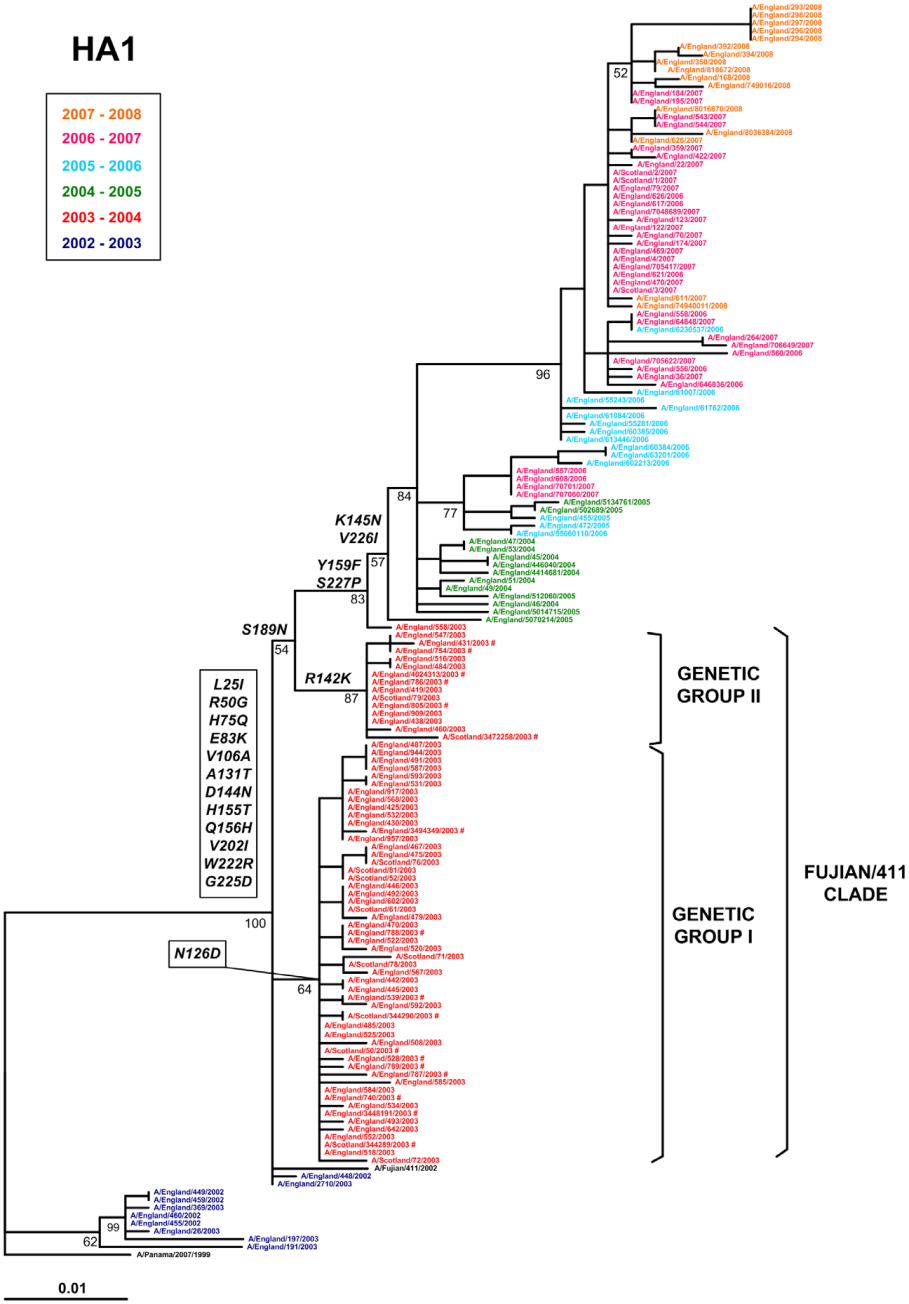
Phylogenetic relationships of partial sequences (nucleotides 346 to 866) of the HA1 coding fragment for A(H3N2) viruses isolated between 2002 and 2008 in the UK. The annotated box indicates the reference colours for viruses isolated in different influenza seasons, between 2002–2003 and 2007–2008. Genetic groups I and II, within the Fujian/411 clade, are indicated with brackets. Viruses from fatal cases are highlighted with #. Sequences from reference viruses A/Panama/2007/99 and A/Fujian/411/2002 were included in the phylogeny. The ML tree was rooted using A/Panama/2007/99 as an outgroup. Branch lengths are drawn to scale. Bootstrap values (>50%) and signature amino acid substitutions seen in viruses from 2003–2004 and 2004–05 are annotated on the nodes.

**Figure 6:**
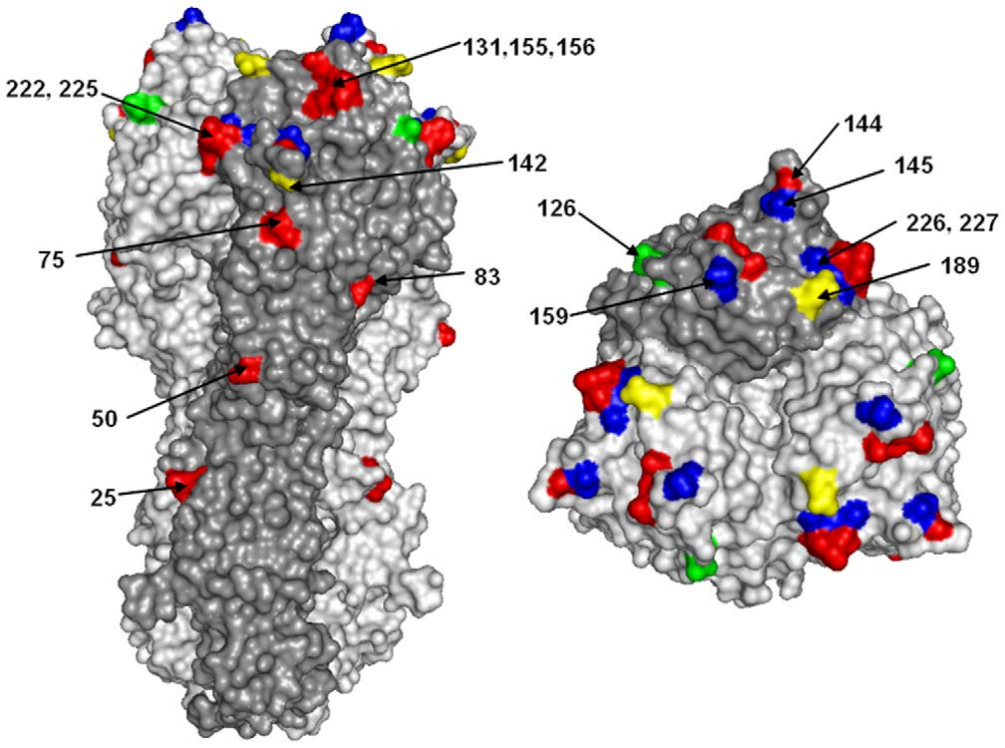
Surface representation of the hemagglutinin hetero-trimer from a seasonal H3N2 strain [**47**]. Location of the amino acids substitutions marking the transition between Panama-like and Fujian-like viruses are highlighted in red; those changes which differentiate viruses isolated since 2004–2005 from Fujian/411-like viruses are coloured in blue; genetic group I-specific changes are highlighted in green and genetic group II-specific changes are marked with yellow.

A total of 159 HA1 sequences were used to build the phylogeny shown in Figure 5: the two major genetic groups from 2003–2004 are marked as I and II. For each season there was a predominant replacement of the older viruses by new ones, with some representatives of each season continuing to survive for one or two seasons further. There was an almost complete predominance of viruses falling within the Fujian/411 clade during 2003–2004 in the UK, all characterized by 12 amino acid substitutions compared to the UK 2002–2003 (Panama/2007-like) viruses (Figure 5 & 6). Of these amino acid changes, 10 were located on the surface of the HA trimer, although two were positioned in the stalk region; and 9 were located in antigenic sites, Within this clade, amino acid substitution N126D defines genetic group I and R142K and S189N, both in antigenic site B, define genetic group II. However, no viruses belonging to these two genetic groups were isolated after that season and the major branch spanning all isolates from 2004–2005 onwards completely diverged from the genetic group II branch. This suggests that viruses representing genetic groups I and II did not survive past the 2003–2004 season. In addition, all viruses characterised since 2004–2005 showed fixation of at least four amino acid substitutions: K145N, Y159F, V226I, S227P, relative to the Fujian/411 clade HA1 genetic groups I and II (Figure 5 & 6). All four changes are exposed on the surface of the globular head of the HA trimer; positions 145 and 159 lie on antigenic sites A and B, whereas positions 226 and 227 are located on the receptor binding site. A/England/558/2003, which clustered with post-2003–2004 viruses, exhibited two of these amino acid changes (Y159F and S227P).

We then compared amino acid substitutions in whole genome products between Fujian/411-like viruses and earlier (Panama/2007-like) viruses (Table 1). Given the unavailability of full genome sequences from UK viruses isolated before 2003–2004, we used instead a set of 40 viruses from GenBank representative of seasons 1999–2000 to 2002–2003 for the comparison (listed in Table S3). Thirteen substitutions were found to distinguish the HA protein of Fujian/411-like viruses from earlier A(H3N2) viruses. An additional 26 substitutions differentiated other gene products, with 11 of them being located in the NA protein. Many of these mutations (marked with * in Table 1) appeared concomitantly in the genome of some viruses isolated in Australia and New York in 2001 and 2002. However, this observation did not include changes seen in the HA sequence.

**Table 1:** Amino acid substitutions in the inferred protein sequences that distinguish UK Fujian/411-like viruses from earlier (Panama/2007-like) viruses.

PB2	PB1	PB1-F2	PA	HA	NP	NA	M1	M2	NS1	NS2
R389K*	T469I*	none	D27N*	L25I	A27S*	K93N	none	none	E71G*	K88R*
I667V*			K262R*	R50G	R98K*	R143G				
			S332T*	H75Q	K103R*	K172R*				
			I348V*	E83K	T146A*	H197D				
			I421V*	A131T	V197I*	E199K				
				H155T		D208N				
				Q156H		R249K				
				S186G		T265I*				
				V202I		K267T				
				W222R		S370L				
				G225D		Q432E*				
				E386G						
				V530A						

Amino acid substitutions are described using the protein sequences of the A/Panama/2007/99 vaccine virus as reference.

* Indicates changes appearing concomitantly in viruses from different countries in 1999–2002 (see Table S3).

## Discussion

An essential question that remains unanswered is whether virus virulence or host-related susceptibility factors have the greater impact on determining the clinical outcome of an influenza infection. In this study we have analysed a set of viruses obtained from a series of paediatric fatal cases with laboratory confirmed A(H3N2) influenza infection, which occurred during the influenza season of 2003–2004 in the UK. Johnson et al [8] described clinical and epidemiological findings from these cases and established some significant facts: (1) 14 of 16 children (88%) for whom health records were available had no pre-existing risk factors; (2) none of the children had received the vaccine for winter 2003–2004; (3) three children (18%) had evidence of bacterial co-infections. This profile indicates a cohort of healthy children who were likely naïve to influenza infection in most cases (65% (11) were under 5 years old) for whom bacterial co-infections may not have contributed significantly to fatal outcome. Therefore we sought to determine whether there were patterns in the phylogenetic relationships or changes in the sequences of the genes and proteins from virus isolates that could be associated with a fatal outcome.

In our study we did not find any clear evidence that single genetic changes in the virus genes or proteins could be associated with a higher, significant occurrence of severe or fatal cases. The majority of substitutions observed correspond to signature amino acid substitutions that differentiated circulating A(H3N2) viruses in the UK during 2003–2004 into genetic groups I and II; a third minor genetic group (III) was also observed. These three genetic groups correlated well with those from 2003–2004 identified in the US [10], as shown by the inclusion of New York strains in the phylogeny presented in Figures 1 to [Fig pone-0033166-g002] 3. The slightly different topology of genetic group III in the HA phylogeny has been previously described as a consequence of a reassortment event that put an HA segment from a 2002–2003 virus into the genomic background of the Fujian/411-like viruses [10], [13]. The existence of similar phylogenetic patterns in our study and in New York reinforces the observation that global migration of influenza viruses is an important component of the seasonal diversity observed every year [14].

Comparison of sequences from 14 pairs of virus isolates vs. primary clinical samples revealed that only one pair differed by a nonsilent mutation in the HA. Host-mediated mutations are a well-known consequence of adaptation to laboratory culture and are a potential source of problems in evolutionary studies [15]. However, finding only one mutated nucleotide out of 183148 (13082 bases per whole genome) would indicate that the effect of cell-culture mediated mutations is irrelevant in our study. Overlapping peaks in the mutated sequence suggested the presence of a mixture of variants in that isolate. The use of deep sequencing would have added valuable information on the diversity of the virus population within each sample. However, as most viruses had only isolates available for sequencing, any attempt to apply deep sequencing to the analysis of intrasample diversity would have been invalidated by the potential selection of variants in tissue culture, therefore not reflecting the true proportion of quasiespecies, if present, in the primary clinical material.

Although the majority of individual gene segments showed identical clustering in phylogenies, there were at least four topological change events in the dendrograms, strongly suggesting evidence of reassortment (Figures 2 & 3), also confirmed by bootscanning and informative sites analysis. This adds further support to the notion that gene reassortment among same-subtype strains is a major driving force for evolution in influenza A virus [16]. When analyzing the worldwide occurrence of variants circulating during 2003–2004 we observed a low frequency of genetic group II viruses (1.5%, Figure 4), compared with a higher frequency in the UK, both for fatal (33 or 42%) and non-fatal (16%) cases, which suggest overrepresentation of genetic group II viruses in the UK in 2003–2004. Although we included the total number of HA1 sequences for influenza A(H3N2) viruses from 2003–2004 available in GenBank for this analysis, we acknowledge the limitations of using GenBank numbers to make epidemiological assumptions, as numbers of sequences are not representative of the population; also, some countries are overrepresented while some do not submit sequence data, so it is possible that genetic group II viruses could have circulated in high frequencies in other geographic locations with poor surveillance system.

Fujian/411-like viruses completely replaced the Panama/2007-like variants circulating in 2002–2003, as shown by analysis of genetic groups I and II in a set of 159 partial HA1 sequences spanning six consecutive influenza seasons from 2002–2003. However, while Fujian/411-like antigenic variants predominated during the 2003–2004 season in the UK, they did not circulate thereafter [17]. In our study, at least 12 amino acid substitutions in the HA1 glycoprotein mark the transition between Panama/2007- and Fujian/411-like viruses. The majority were located on the surface of the HA trimer and within one of the five antigenic sites proposed for H3 haemagglutinin [18], [19], therefore showing potential for antigenic variation, although only H155T and Q156H were actually responsible for the antigenic drift [20]. Four further amino acid substitutions in HA1 differentiate the Fujian/411-like viruses from viruses isolated after 2003–2004. All of them were also exposed on the surface of the globular head, either related to antigenic sites A or B, or located in the receptor binding site. In our study, the Fujian/411-like viruses exhibited substitutions W222R and G225D when compared to the previous Panama/2007-like variants. Recently, substitution D225G in the HA (D222G by H1 numbering) of pandemic influenza A(H1N1) 2009 viruses has been implicated in higher severity and fatal cases [21]. This substitution has been shown to induce alterations in the receptor binding site, increasing the affinity of these viruses for receptors expressed on ciliated bronchial epithelial cells and on epithelia within the lung [22]. Position 226 (H3 numbering) has also been implicated in changes of receptor binding specificity, cell tropism and host range restriction for several human and animal influenza subtypes [23], [24], [25], [26]. In summary, HA changes distinguishing the transition between Panama/2007-like, Fujian/411-like and later viruses have enough potential for altering biological properties such as antigenicity, receptor binding affinity or others which in turn might affect virus virulence.

An additional 26 substitutions were shown to distinguish the remaining gene products of Fujian/411-like viruses from earlier (Panama/2007-like) viruses. Eighteen of them were already detected concurrently in viruses isolated in 2001 and 2002, suggesting that the genomic background of the Fujian/411-like viruses was already circulating at that time. However, these viruses did not have the HA changes seen in the Fujian/411-like viruses, an observation that corresponds with other reports suggesting that the HA gene of Fujian/411-like viruses was only placed by reassortment into the genomic background of the Fujian/411-like viruses shortly before the 2003–2004 season [10], [13]. It has also been suggested that interaction among viral proteins may play a key role in the fitness and virulence of a virus [10]. Some of the genomic substitutions observed in the Fujian/411-like viruses were related to host range specificity. For instance, substitution I667V in PB2 represents a human-to-avian specific change [27]. Substitutions V421I(PB1) and T9N(PB2) were described as human-like changes in H5N1 viruses with increased pathogenicity in mice [28]. The biological relevance of substitutions I421V and N9T (characteristic of genetic group I) seen in the Fujian/411-like viruses is unknown. Substitutions at positions 27, 103, 146 and 197 in NP are located in known virus cytotoxic T lymphocyte (CTL) epitopes [29], [30], [31] and they may confer on Fujian/411-like viruses a higher efficiency of escape from CTL-mediated immune responses. Substitutions at positions 172, 199 and 370 in NA are located in antigenic sites [32], while the K93N substitution introduces an additional potential glycosylation site; all/some of these may contribute to the antigenic drift seen in Fujian/411-like viruses. Many of these substitutions (marked with * in Table 1) have been seen concomitantly in the genome of some A(H3N2) viruses from before 2003–2004. A search in the NCBI Influenza Virus Resource through all the available A(H3N2) genome sequences (>2,000) showed that viruses exhibiting these substitutions were seen from seasons 2001–2002 to 2003–2004 disappearing thereafter, consistent with the replacement of the Fujian/411-like viruses in 2004–2005 in a genome-wide sweep, as previously described [3].

While Fujian/411-like viruses exhibited a unique gene constellation and carried HA amino acid substitutions that caused significant antigenic drift [33] and replacement of all other A(H3N2) viruses circulating that season, no specific genetic changes and consequent amino acid substitutions could be related to severe or fatal clinical outcomes. On this basis, we conclude that host-related susceptibility was the major determinant of severe illness or death due to infection by Fujian/411-like viruses in 2003–2004. It is likely that, if any virus feature contributed to a more severe outcome, this occurred in the context of naïve children undergoing what was likely to be their first influenza infection.

Clinical incidences of influenza-like illness (ILI) in young age groups were shown to be at their highest in the years when novel antigenic drift variants emerged [8]. Similarly, studies of mortality in children associated with pandemic influenza A(H1N1) 2009 revealed the highest mortality rate in children aged less than 1 year and a substantial proportion of children with no pre-existing disorders (27%) who rapidly deteriorated and died [34], [35]. Seven out of 17 paediatric deaths in the UK in 2003–2004 occurred suddenly with no previous illness or within a day of the onset of symptoms, with no obvious link to a predominance of genetic groups I or II viruses among these cases. Overall, we found no consistent relationship between the genetic features of these viruses and the main clinical findings observed for the UK paediatric fatal cases (Table S2).

The contribution of host factors to the outcome of an influenza infection is supported by our finding that the same, genetically identical virus (represented by A/England/740/2003) could cause death in a child while causing uncomplicated respiratory illness in one family contact (a sibling of unknown age) and two non-related adult contacts. Factors related to the infecting-dose of virus could not be determined. Innate immune response, preexisting adaptive immunity, genetic background [36], [37] and even environmental conditions surrounding host and virus (reviewed in [38]) are all factors that can contribute to virus pathogenesis.

Limitations of the present study include the probable underestimate of the number of paediatric death cases during the 2003–2004 influenza season as we have studied only those reported to the HPA and laboratory-confirmed. Since this time, reporting mechanism of fatal cases has been strengthened. Also, the whole genome sequence data available outside the UK was biased to specific countries and this may have altered the interpretation. However, this study reinforces the conclusion that the virulence of an influenza virus results from a complex interplay between polygenic virus virulence factors, the host immune response and host genetic factors. Any difference in virulence between genetic groups I and II viruses could be tested in animal models by using recombinant viruses generated through reverse genetic systems. It is possible that the Fujian/411-like viruses (genetic groups I and II) were all more virulent than previously circulating viruses. Alternatively, if any virus factor was contributing to a higher virulence, its relative impact could have been substantial in the context of a highly susceptible host. Additional studies are needed to better identify host genetic determinants of severe influenza infection, perhaps focussing on human genes responsible for the development of an atypical antiviral innate immune response. This could be a critical factor that leads to severe illness and death in naïve hosts. The matching of virus genomics with clinical and epidemiological findings is an important tool for studying highly mutable pathogens, such as influenza A virus. The methodology carried out in this study will be useful in the analysis of seasonal epidemics of influenza and also in the study of the recently emerged pandemic influenza A(H1N1) 2009 virus.

## Materials and Methods

### Ethics statement

This study was conducted as part of a public health inquiry by the Health Protection Agency and Health Protection Scotland into paediatric deaths, where it was agreed that ethical approval for linking personal clinical outcome information to virus characterisation was not required.

### Fatal and control cases

Seventeen fatal cases of laboratory-confirmed influenza A in children under eighteen years were reported to HPA between September and December 2003. The median age was two years (range 4 months – 17 years); eleven (64%) were under five years of age and ten (58%) were females. Further clinical findings are described in Johnson et al [8]. Three control cases were selected per fatal case, on the basis of matching age, week of infection and geographical location. HPA influenza surveillance relies on the timely collection of clinical and virological data across the UK. Information sources have been described previously [33]. Overall, over 80% of reported laboratory-confirmed cases have respiratory specimens referred to National Reference Laboratory.

### Viruses

Respiratory samples and tissue biopsy samples were received at HPA for laboratory confirmation of A(H3N2) influenza virus infection and further characterisation of the infecting viruses. Original material was inoculated onto either Madin-Darby canine kidney cells (MDCK) or the SIAT1 derivative [39]. Virological data is described with more details elsewhere [33].

### Whole genome sequencing

Virus RNA was extracted from either aliquots of original respiratory/biopsy material or from virus isolates using a Qiagen viral RNA mini kit (Qiagen Ltd, Crawley, West Sussex, England). Complete genomes were amplified using a one-step RT-PCR procedure employing Superscript III RT and Platinum *Pfx*, a proof-reading DNA polymerase (Invitrogen Ltd, Paisley, England). RT-PCR products were purified using QIAquick gel extraction and PCR purification kit (Qiagen). Sequences were obtained by a gene-walking approach using a commercial sequencing service (GATC Biotech Ltd, Cambridge, England). Specific primers for amplification and sequencing are described in Table S1. Genbank accession numbers range from CY087969 to CY088568 for virus isolate sequences and from CY107071 to CY107182 for original material sequences.

### Phylogenetic analyses

Raw sequencing data was edited and assembled into continuous gene sequences (contigs) covering the entire ORF of each gene using Sequencher 4.9 (http://www.genecodes.com).

Nucleotide sequences for each gene segment were aligned using ClustalX 1.81 [40]. Alignments were edited with BioEdit 7.0.5.3 [41]. Maximum Likelihood (ML) phylogenetic trees of each gene segment were inferred using the PAUP 4.0 package (Swofford DL; Sinauer Associates). The best-fit model of nucleotide substitution was identified using Modeltest [42] as the general-time reversible GTR+Γ_4_ model for most genes, except for NP (GTR+I), NS (GTR) and M (HKY85+Γ_4_). Nucleotide frequencies, transition-transversion ratio, proportion of invariable sites and gamma distribution of among-site rate variation were estimated from the empirical data. In all cases, ML trees were determined through a heuristic search using the tree bisection-reconnection branch-swapping algorithm.

Bootstrap analysis was performed through the Neighbor Joining algorithm with 1,000 replicates, incorporating the ML substitution model previously determined for each gene with Modeltest.

Two large datasets, the set of 1,893 sequences used to generate the phylogeny presented in Figure 4 and the 70 sequences formed by alphabetic concatenation of influenza coding regions (Figure 1) were aligned using a parallel implementation of ClustalW, ClustalW-MPI [43]. Phylogenetic trees from these alignments were inferred using a rapid parallelised ML inference method; RAxML [44]. RAxML utilises the general-time reversible (GTR) model of nucleotide substitution and performs heuristic ML searches using maximum parsimony trees as starting points. The trees were created using 200 independent inferences and 1,000 bootstrap replicates.

Identification of recombination/reassortment breakpoints in the concatenated whole genome sequences through bootscanning and informative site analysis was performed using Simplot v3.2 [45]

Potential N-glycosylation sites in the deduced amino acid sequences were predicted using NetNGlyc version 1.0 [46].

### Structural analysis

A model of the HA influenza trimer was generated using an X-ray crystallographic structure (PDB code: 2VIU [47]) downloaded from the Protein Data Bank website [48]. The structure was manipulated and presented using PyMOL. One monomer of HA trimer was highlighted to indicate the arrangement of monomers within the overall trimers. The structural position of amino acid variants discriminating between Panama/Fujian/later strains and the positions specific to genetic groups I and II were highlighted and labelled on the trimeric structure.

## Supporting Information

Figure S1 **Bootscan analysis for reassortant UK viruses.** A) A/England/431/2003; B) A/England/585/2003; C) A/England/558/2003; D) A/England/567/2003; E) A/Scotland/78/2003.
(TIF)

Table S1 **Primers for whole genome sequencing of Influenza A(H3N2) viruses.** Primers for gene fragment amplification and sequencing are highlighted in bold.
(DOC)

Table S2 **Relationship between phylogenetic clustering of viruses from fatal cases and main clinical findings (described with more detail in** [**8**] **).**
(DOC)

Table S3 **Set of A(H3N2) viruses selected from GenBank and used for the amino acid substitution comparison featured in** **Table 1** **.**
(DOC)

Text S1 **FindSites analysis of the reassortant UK viruses.**
(RTF)
