# Supplementary figures and images for: Fatal Cases of Influenza A(H3N2) in Children: Insights from Whole Genome Sequence Analysis
Source: PLoS One. 2012 Mar 6;7(3):e33166. doi: 10.1371/journal.pone.0033166 (PMC3295814; doi:10.1371/journal.pone.0033166)

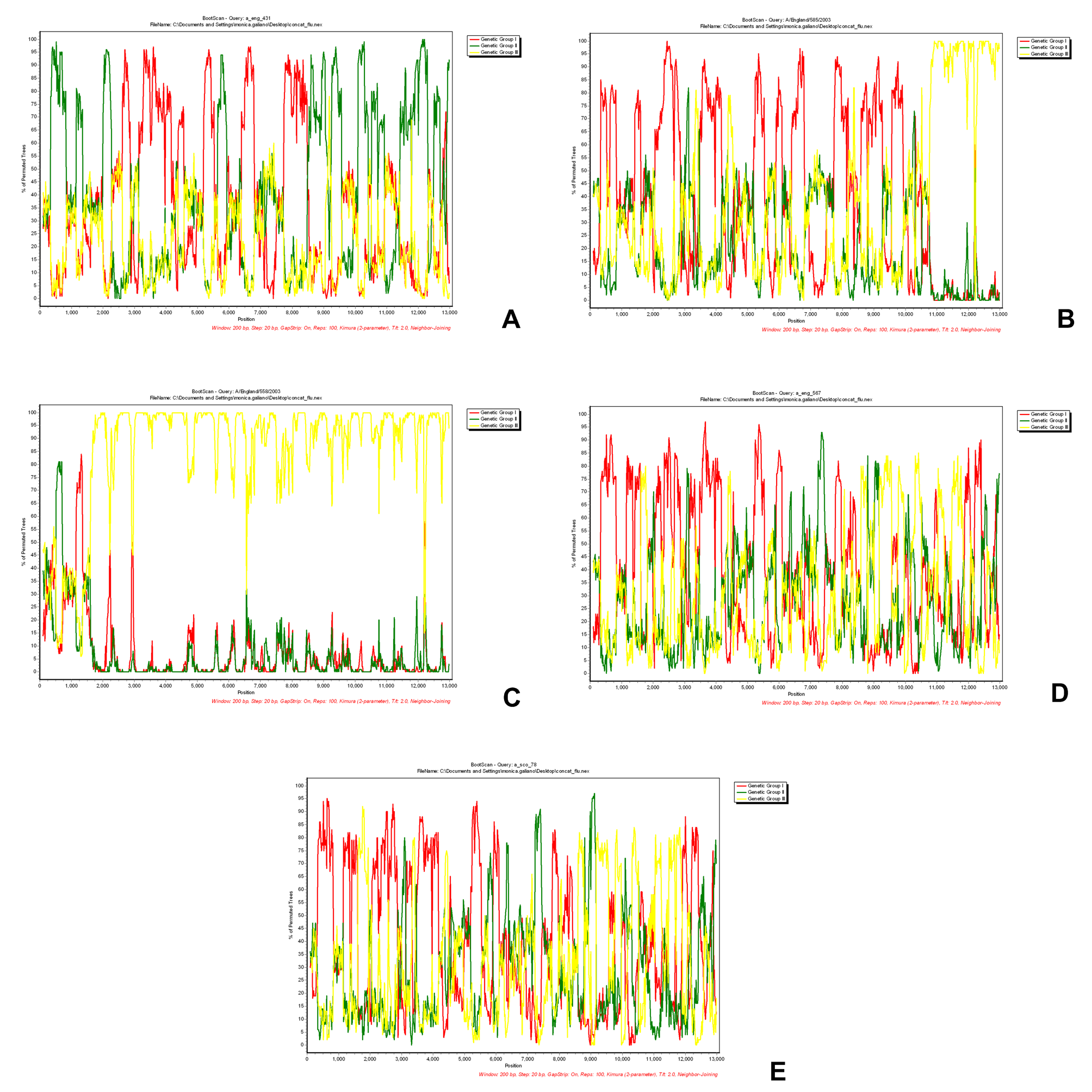

Supplement: Figure S1 — Bootscan analysis for reassortant UK viruses. A) A/England/431/2003; B) A/England/585/2003; C) A/England/558/2003; D) A/England/567/2003; E) A/Scotland/78/2003. (TIF) [file pone.0033166.s001.tif]
