# Supplementary material for: Fatal Cases of Influenza A(H3N2) in Children: Insights from Whole Genome Sequence Analysis
Source: PLoS One. 2012 Mar 6;7(3):e33166. doi: 10.1371/journal.pone.0033166 (PMC3295814; doi:10.1371/journal.pone.0033166)
Supplement: Table S2 — Relationship between phylogenetic clustering of viruses from fatal cases and main clinical findings (described with more detail in [8] ). (DOC) [file pone.0033166.s003.doc]

Table S2

| Case N° | Virus* | Genetic  Group | Clinical findings |
| --- | --- | --- | --- |
| 1 | A/Scotland/344290/2003 (only HA1 sequence) | I | Several days of flu-like symptoms with vomiting and diarrhoea |
| 2 | A/Scotland/344289/2003 (only HA1 sequence) | I | Sudden death. Cerebral oedema |
| 3 | A/Scotland/50/2003 | I | Several days of vomiting. Collapse. Septicaemia due to *S. aureus* |
| 4 | A/England/431/2003 | II | Sudden death |
| 5 | A/England/3448191/2003 (only HA1 sequence) | I | Mild illness, hypothermia and collapse |
| 6 | A/England/740/2003 | I | Several days of fever, chest infection, cardiac arrest |
| 7 | A/England/786/2003 | II | 24h sore throat, temperature, sudden collapse |
| 8 | A/England/754/2003 | II | Sudden death |
| 9 | A/England/539/2003 | I | Fever, cough, cold. Collapse |
| 10 | A/England/789/2003 | I | Sudden death |
| 11 | A/England/788/2003 | I | Sudden death |
| 12 | A/England/787/2003 | I | Cough, apnoea |
| 13 | A/England/805/2003 | II | Fever, tachypnea |
| 14 | A/England/3494349/2003 (only HA1 sequence) | I | 3 days croup-like illness. *S. pneumoniae* septicaemia |
| 15 | A/Scotland/3472258/2003 (only HA1 sequence) | II | Flu-like illness, lethargy. *Streptococcal* pneumonia |
| 16 | A/England/528/2003 | I | Mild respiratory illness, then haemorrhagic pneumonia |
| 17 | A/England/4024313/2003 | II | 24h temperature, lethargy. Brain oedema |

*For five clinical specimens, virus isolation was not successful and sequence encoding the HA1 ORF only was determined.
