# Supplementary material for: Fatal Cases of Influenza A(H3N2) in Children: Insights from Whole Genome Sequence Analysis
Source: PLoS One. 2012 Mar 6;7(3):e33166. doi: 10.1371/journal.pone.0033166 (PMC3295814; doi:10.1371/journal.pone.0033166)
Supplement: Table S3 — Set of A(H3N2) viruses selected from GenBank and used for the amino acid substitution comparison featured in Table 1 . (DOC) [file pone.0033166.s004.doc]

Table S3

| No | Virus | GenBank accession number |
| --- | --- | --- |
| 1 | A/Auckland/593/2000 | CY022517 - CY022524 |
| 2 | A/Auckland/600/2000 | CY023042 - CY023049 |
| 3 | A/Auckland/611/2002† | CY022189 - CY022196 |
| 4 | A/Auckland/614/2002† | CY022205 - CY022212 |
| 5 | A/Canterbury/04/2002† | CY008003 - CY008010 |
| 6 | A/Canterbury/101/2000 | CY008844 - CY008851 |
| 7 | A/Canterbury/140/2001 | CY009588 - CY009595 |
| 8 | A/Canterbury/35/2002† | CY007675 - CY007682 |
| 9 | A/Canterbury/43/2001 | CY009572 - CY009579 |
| 10 | A/Canterbury/76/2002† | CY008332 - CY008339 |
| 11 | A/New South Wales/17/99 | CY016635 - CY016642 |
| 12 | A/New South Wales/19/99 | CY020205 - CY020212 |
| 13 | A/New York/106/2002† | CY000489 - CY000496 |
| 14 | A/New York/134/2002† | CY000441 - CY000448 |
| 15 | A/New York/147/99 | CY000593 - CY000600 |
| 16 | A/New York/156/2000 | CY001397 - CY001404 |
| 17 | A/New York/157/99 | CY000825 - CY000832 |
| 18 | A/New York/175/2000 | CY000705 - CY000712 |
| 19 | A/New York/177/99 | CY000721 - CY000728 |
| 20 | A/New York/261/99 | CY002560 - CY002567 |
| 21 | A/New York/265/99 | CY001616 - CY001623 |
| 22 | A/New York/288/99 | CY001776 - CY001783 |
| 23 | A/New York/318/99 | CY001848 - CY001855 |
| 24 | A/New York/336/99 | CY001912 - CY001919 |
| 25 | A/New York/404/2002† | CY003104 - CY003111 |
| 26 | A/New York/419/2002† | CY003208 - CY003215 |
| 27 | A/New York/451/99 | CY003584 - CY003591 |
| 28 | A/New York/77/2001† | CY002328 - CY002335 |
| 29 | A/New York/83/2001† | CY000185 - CY000192 |
| 30 | A/New York/85/2001† | CY000385 - CY000392 |
| 31 | A/Panama/2007/99 | DQ487333 - DQ487340 |
| 32 | A/Queensland/1/2000 | CY017259 - CY017266 |
| 33 | A/Queensland/12/2001† | CY017491 - CY017498 |
| 34 | A/Queensland/16/2001† | CY017507 - CY017514 |
| 35 | A/Queensland/28/2002† | CY017917 - CY017924 |
| 36 | A/Queensland/30/2002† | CY018965 - CY018972 |
| 37 | A/South Australia/81/2000 | CY021781 - CY021788 |
| 38 | A/Waikato/16/2000 | CY013389 - CY013396 |
| 39 | A/Waikato/20/2000 | CY013064 - CY013071 |
| 40 | A/Waikato/25/2002† | CY013088 - CY013095 |
| 41 | A/Western Australia/10/2000 | CY016491 - CY016498 |

† Viruses with substitutions marked as * and described in Table 1
