# Supplementary material for: Fatal Cases of Influenza A(H3N2) in Children: Insights from Whole Genome Sequence Analysis
Source: PLoS One. 2012 Mar 6;7(3):e33166. doi: 10.1371/journal.pone.0033166 (PMC3295814; doi:10.1371/journal.pone.0033166)
Supplement: Text S1 — FindSites analysis of the reassortant UK viruses. (RTF) [file pone.0033166.s005.rtf]

FindSites analysis (Simplot) for A/England/431/2003

Using the letters A, B, C, and D:
A =  A/England/431/2003
B =  Genetic Group I
C =  Genetic Group II
D =  Genetic Group III

There are 3 possible bifurcating trees:
     1          2         3
  A     C    A     B   A     B
   \___/      \___/     \___/
   /   \      /   \     /   \
  B     D    C     D   D     C


The following sites support the indicated trees:

Total sites: 56

  pos   A    B    C    D    tree#
  ---   -    -    -    -    -----
  426   C    T    C    T    2
  473   A    G    A    G    2
  614   A    G    A    G    2
  723   C    T    C    T    2
 1257   G    A    G    A    2
 2082   T    C    T    C    2
 2136   A    C    A    C    2
 2169   T    C    T    C    2
 2713   G    G    A    A    1
 2776   C    C    T    T    1
 3222   G    G    A    A    1
 3375   G    G    A    A    1
 3390   A    A    G    G    1
 3564   T    T    C    C    1
 3653   T    T    C    C    1
 3846   T    T    C    C    1
 4077   T    T    C    C    1
 4464   C    C    T    T    1
 5301   A    A    G    G    1
 5334   A    A    G    G    1
 5430   G    G    A    A    1
 5731   A    G    A    G    2
 5841   T    C    T    C    2
 6501   C    C    T    T    1
 6591   C    C    T    T    1
 6692   C    C    T    T    1
 6701   C    C    T    T    1
 7829   A    A    C    C    1
 7862   A    A    G    G    1
 8024   G    G    A    A    1
 8153   T    T    C    C    1
 8249   T    T    C    C    1
 8428   A    A    G    G    1
 8579   T    C    T    C    2
 8900   G    A    G    A    2
 9038   A    G    A    G    2
 9146   G    A    A    G    3
 9284   A    G    A    G    2
 9359   G    A    G    A    2
 9476   G    A    G    A    2
10190   T    C    T    C    2
10196   G    A    G    A    2
10310   A    G    A    G    2
10616   T    C    T    C    2
10832   A    G    A    G    2
11516   A    G    A    G    2
11675   A    G    A    G    2
11894   T    A    T    A    2
11999   C    T    C    T    2
12116   C    T    C    T    2
12137   C    T    C    T    2
12191   G    A    G    A    2
12206   C    T    C    T    2
12596   C    T    C    T    2
12983   A    G    A    G    2
13040   T    C    T    C    2

FindSites analysis (Simplot) for A/England/585/2003

Using the letters A, B, C, and D:
A =  A/England/585/2003
B =  Genetic Group I
C =  Genetic Group II
D =  Genetic Group III

There are 3 possible bifurcating trees:
     1          2         3
  A     C    A     B   A     B
   \___/      \___/     \___/
   /   \      /   \     /   \
  B     D    C     D   D     C


The following sites support the indicated trees:

Total sites: 72

  pos   A    B    C    D    tree#
  ---   -    -    -    -    -----
  424   G    G    A    A    1
  733   C    C    A    A    1
 1518   G    G    A    A    1
 2157   A    A    G    G    1
 2352   G    G    A    A    1
 2457   A    A    G    G    1
 2507   C    C    T    T    1
 2556   G    G    A    A    1
 2776   C    C    T    T    1
 3564   T    T    C    C    1
 3653   T    T    C    C    1
 3846   T    T    C    C    1
 4077   T    T    C    C    1
 5334   A    A    G    G    1
 5430   G    G    A    A    1
 5993   A    A    C    C    1
 6501   C    C    T    T    1
 6692   C    C    T    T    1
 6701   C    C    T    T    1
 7829   A    A    C    C    1
 7862   A    A    G    G    1
 8024   G    G    A    A    1
 8249   T    T    C    C    1
 8295   C    T    T    C    3
 8930   T    T    C    C    1
 9179   C    C    T    T    1
 9674   C    C    T    T    1
 9836   T    T    C    C    1
10406   C    C    T    T    1
10436   A    G    G    A    3
10862   G    A    A    G    3
10910   G    A    A    G    3
10967   C    T    T    C    3
11003   T    C    C    T    3
11015   T    C    C    T    3
11114   C    A    A    C    3
11126   G    A    A    G    3
11132   T    C    C    T    3
11168   C    T    T    C    3
11345   G    A    A    G    3
11369   A    G    G    A    3
11507   T    C    C    T    3
11570   C    T    T    C    3
11612   C    T    T    C    3
11699   A    G    G    A    3
11750   A    G    G    A    3
11792   T    C    C    T    3
11821   A    G    G    A    3
11825   G    A    A    G    3
11861   G    A    A    G    3
11968   G    A    A    G    3
11969   A    G    G    A    3
11999   C    T    C    T    2
12071   A    G    G    A    3
12077   T    C    C    T    3
12083   G    A    A    G    3
12101   G    A    A    G    3
12326   C    T    T    C    3
12374   T    A    A    T    3
12395   T    C    C    T    3
12404   G    A    A    G    3
12554   C    T    T    C    3
12557   C    T    T    C    3
12570   A    G    G    A    3
12617   C    T    T    C    3
12635   C    T    T    C    3
12708   C    T    T    C    3
12801   A    G    G    A    3
12881   G    A    A    G    3
12944   T    C    C    T    3
12965   G    A    A    G    3
12980   C    T    T    C    3


FindSites analysis (Simplot) for A/England/558/2003

Using the letters A, B, C, and D:
A =  A/England/558/2003
B =  Genetic Group I
C =  Genetic Group II
D =  Genetic Group III

There are 3 possible bifurcating trees:
     1          2         3
  A     C    A     B   A     B
   \___/      \___/     \___/
   /   \      /   \     /   \
  B     D    C     D   D     C


The following sites support the indicated trees:

Total sites: 201

  pos   A    B    C    D    tree#
  ---   -    -    -    -    -----
 1260   A    A    G    G    1
 1716   C    A    A    C    3
 1770   G    A    A    G    3
 1854   T    C    C    T    3
 1917   A    G    G    A    3
 1965   A    G    G    A    3
 2004   G    A    A    G    3
 2025   G    A    A    G    3
 2049   T    A    A    T    3
 2121   T    C    C    T    3
 2157   A    A    G    G    1
 2184   T    C    C    T    3
 2353   G    A    A    G    3
 2476   C    T    T    C    3
 2540   G    A    A    G    3
 2566   T    G    G    T    3
 2575   C    G    G    C    3
 2685   G    A    A    G    3
 2710   T    G    G    T    3
 2725   T    C    C    T    3
 2727   C    T    T    C    3
 2746   A    G    G    A    3
 2775   A    G    G    A    3
 2783   T    G    G    T    3
 2808   G    A    A    G    3
 3063   A    G    G    A    3
 3086   T    G    G    T    3
 3123   T    C    C    T    3
 3144   G    A    A    G    3
 3163   C    T    T    C    3
 3168   G    A    A    G    3
 3173   A    G    G    A    3
 3243   G    T    T    G    3
 3305   T    G    G    T    3
 3324   C    A    A    C    3
 3330   G    A    A    G    3
 3342   A    G    G    A    3
 3393   T    C    C    T    3
 3452   C    T    T    C    3
 3576   C    T    T    C    3
 3577   A    G    G    A    3
 3670   T    C    C    T    3
 3672   G    A    A    G    3
 3777   A    T    T    A    3
 3798   T    C    C    T    3
 3813   C    A    A    C    3
 3828   G    A    A    G    3
 3855   C    A    A    C    3
 3891   T    C    C    T    3
 3948   A    G    G    A    3
 3968   T    G    G    T    3
 4047   C    T    T    C    3
 4147   G    T    T    G    3
 4180   A    C    C    A    3
 4242   G    A    A    G    3
 4287   A    G    G    A    3
 4298   A    G    G    A    3
 4347   G    A    A    G    3
 4361   G    A    A    G    3
 4362   G    A    A    G    3
 4376   A    G    G    A    3
 4404   C    T    T    C    3
 4431   C    T    T    C    3
 4476   A    G    G    A    3
 4488   T    C    C    T    3
 4581   T    C    C    T    3
 4584   C    T    T    C    3
 4657   G    A    A    G    3
 4860   G    A    A    G    3
 5001   A    G    G    A    3
 5004   A    G    G    A    3
 5022   T    G    G    T    3
 5118   A    C    C    A    3
 5139   A    G    G    A    3
 5196   G    A    A    G    3
 5199   C    T    T    C    3
 5285   C    T    T    C    3
 5340   C    T    T    C    3
 5341   A    G    G    A    3
 5379   T    C    C    T    3
 5445   G    T    T    G    3
 5449   A    C    C    A    3
 5508   T    A    A    T    3
 5709   T    C    C    T    3
 5754   A    G    G    A    3
 5775   A    G    G    A    3
 5777   A    G    G    A    3
 5810   T    C    C    T    3
 5892   G    A    A    G    3
 5907   T    C    C    T    3
 5976   T    C    C    T    3
 6117   A    G    G    A    3
 6300   A    G    G    A    3
 6310   A    G    G    A    3
 6316   C    T    T    C    3
 6322   A    G    G    A    3
 6349   A    G    G    A    3
 6456   G    A    A    G    3
 6461   G    A    A    G    3
 6680   A    G    G    A    3
 6803   C    T    T    C    3
 6905   T    C    C    T    3
 6914   A    G    G    A    3
 7013   T    C    C    T    3
 7037   A    G    G    A    3
 7162   A    G    G    A    3
 7202   T    C    C    T    3
 7287   T    C    C    T    3
 7371   T    A    A    T    3
 7382   G    A    A    G    3
 7419   A    G    G    A    3
 7424   A    G    G    A    3
 7638   A    G    G    A    3
 7782   C    T    T    C    3
 7895   G    A    A    G    3
 8009   G    A    A    G    3
 8135   T    A    A    T    3
 8141   A    G    G    A    3
 8144   C    A    A    C    3
 8270   T    C    C    T    3
 8295   C    T    T    C    3
 8297   A    G    G    A    3
 8309   A    G    G    A    3
 8333   A    T    T    A    3
 8342   G    A    A    G    3
 8552   G    T    T    G    3
 8681   G    A    A    G    3
 8831   T    G    G    T    3
 8897   C    T    T    C    3
 8903   T    C    C    T    3
 8945   A    G    G    A    3
 8990   A    T    T    A    3
 9008   A    G    G    A    3
 9146   G    A    A    G    3
 9278   G    A    A    G    3
 9392   A    G    G    A    3
 9464   T    C    C    T    3
 9482   T    C    C    T    3
 9656   T    C    C    T    3
 9722   T    C    C    T    3
 9863   C    T    T    C    3
 9934   C    T    T    C    3
 9944   A    G    G    A    3
 9992   G    A    A    G    3
10022   A    C    C    A    3
10082   C    T    T    C    3
10172   C    T    T    C    3
10285   G    A    A    G    3
10304   A    G    G    A    3
10328   T    C    C    T    3
10346   C    T    T    C    3
10349   T    C    C    T    3
10383   A    G    G    A    3
10410   C    T    T    C    3
10436   A    G    G    A    3
10442   G    A    A    G    3
10553   C    A    A    C    3
10653   A    G    G    A    3
10745   G    A    A    G    3
10862   G    A    A    G    3
10910   G    A    A    G    3
10967   C    T    T    C    3
11003   T    C    C    T    3
11015   T    C    C    T    3
11114   C    A    A    C    3
11126   G    A    A    G    3
11132   T    C    C    T    3
11168   C    T    T    C    3
11345   G    A    A    G    3
11369   A    G    G    A    3
11507   T    C    C    T    3
11570   C    T    T    C    3
11612   C    T    T    C    3
11699   A    G    G    A    3
11708   G    T    T    G    3
11750   A    G    G    A    3
11792   T    C    C    T    3
11821   A    G    G    A    3
11825   G    A    A    G    3
11861   G    A    A    G    3
11968   G    A    A    G    3
11969   A    G    G    A    3
12071   A    G    G    A    3
12077   T    C    C    T    3
12083   G    A    A    G    3
12101   G    A    A    G    3
12326   C    T    T    C    3
12374   T    A    A    T    3
12395   T    C    C    T    3
12404   G    A    A    G    3
12554   C    T    T    C    3
12557   C    T    T    C    3
12570   A    G    G    A    3
12617   C    T    T    C    3
12635   C    T    T    C    3
12708   C    T    T    C    3
12881   G    A    A    G    3
12944   T    C    C    T    3
12965   G    A    A    G    3
12980   C    T    T    C    3
12983   G    G    A    A    1

FindSites analysis (Simplot) for A/England/567/2003

Using the letters A, B, C, and D:
A =  A/England/567/2003
B =  Genetic Group I
C =  Genetic Group II
D =  Genetic Group III

There are 3 possible bifurcating trees:
     1          2         3
  A     C    A     B   A     B
   \___/      \___/     \___/
   /   \      /   \     /   \
  B     D    C     D   D     C


The following sites support the indicated trees:

Total sites: 32

  pos   A    B    C    D    tree#
  ---   -    -    -    -    -----
  424   G    G    A    A    1
  614   G    G    A    A    1
  733   C    C    A    A    1
 1248   G    G    A    A    1
 1518   G    G    A    A    1
 1716   C    A    A    C    3
 2157   A    A    G    G    1
 2457   A    A    G    G    1
 2776   C    C    T    T    1
 3222   G    G    A    A    1
 3564   T    T    C    C    1
 3653   T    T    C    C    1
 3846   T    T    C    C    1
 4077   T    T    C    C    1
 5334   A    A    G    G    1
 5430   G    G    A    A    1
 5993   A    A    C    C    1
 7337   C    T    C    T    2
 7862   A    A    G    G    1
 8900   G    A    G    A    2
 8903   T    C    C    T    3
 9278   G    A    A    G    3
 9464   T    C    C    T    3
 9863   C    T    T    C    3
10304   A    G    G    A    3
10310   A    G    A    G    2
10410   C    T    T    C    3
11132   T    C    C    T    3
11570   C    T    T    C    3
11972   T    T    C    C    1
12302   A    A    G    G    1
12983   A    G    A    G    2


FindSites analysis (Simplot) for A/England/567/2003

Using the letters A, B, C, and D:
A =  A/England/567/2003
B =  Genetic Group I
C =  Genetic Group II
D =  Genetic Group III

There are 3 possible bifurcating trees:
     1          2         3
  A     C    A     B   A     B
   \___/      \___/     \___/
   /   \      /   \     /   \
  B     D    C     D   D     C


The following sites support the indicated trees:

Total sites: 35

  pos   A    B    C    D    tree#
  ---   -    -    -    -    -----
  424   G    G    A    A    1
  614   G    G    A    A    1
  733   C    C    A    A    1
 1248   G    G    A    A    1
 1518   G    G    A    A    1
 1716   C    A    A    C    3
 1854   T    C    C    T    3
 2157   A    A    G    G    1
 2457   A    A    G    G    1
 2776   C    C    T    T    1
 3222   G    G    A    A    1
 3564   T    T    C    C    1
 3653   T    T    C    C    1
 3846   T    T    C    C    1
 4077   T    T    C    C    1
 5334   A    A    G    G    1
 5430   G    G    A    A    1
 5993   A    A    C    C    1
 7337   C    T    C    T    2
 7862   A    A    G    G    1
 8900   G    A    G    A    2
 8903   T    C    C    T    3
 9038   A    G    A    G    2
 9278   G    A    A    G    3
 9464   T    C    C    T    3
 9863   C    T    T    C    3
10304   A    G    G    A    3
10310   A    G    A    G    2
10410   C    T    T    C    3
11132   T    C    C    T    3
11570   C    T    T    C    3
11821   A    G    G    A    3
11972   T    T    C    C    1
12302   A    A    G    G    1
12983   A    G    A    G    2
